# Impact of Dose of Combined Conventional and Robotic Therapy on Upper Limb Motor Impairments and Costs in Subacute Stroke Patients: A Retrospective Study

**DOI:** 10.3389/fneur.2022.770259

**Published:** 2022-02-09

**Authors:** Ophélie Pila, Typhaine Koeppel, Anne-Gaëlle Grosmaire, Christophe Duret

**Affiliations:** Centre de Rééducation Fonctionnelle Les Trois Soleils, Unité de Neurorééducation, Boissise-Le-Roi, France

**Keywords:** stroke rehabilitation, robotics, motor recovery, upper limb, treatment dose

## Abstract

**Introduction:**

Robot-based training integrated into usual care might optimize therapy productivity and increase treatment dose. This retrospective study compared two doses of an upper limb rehabilitation program combining robot-assisted therapy and occupational therapy on motor recovery and costs after stroke.

**Methods:**

Thirty-six subacute stroke patients [Fugl-Meyer Assessment (FMA) score 32 ± 12 points; mean ± SD] underwent a combined program of 29 ± 3 sessions of robot-assisted therapy and occupational therapy. Scheduled session time for the higher dose group (HG) was 90 min (two 45-min sessions; *n* = 14) and for the lower dose group (LG) was 60 min (two 30-min sessions; *n* = 22). Pre-/post-treatment change in FMA score (ΔFMA, %), actual active time (min), number of movements and number of movements per minute per robot-assisted therapy session were compared between groups. The costs of the combined programs were also analyzed.

**Results:**

ΔFMA did not differ significantly between groups; the HG improved by 16 ± 13 % and the LG by 11 ± 8%. A between-group difference was found for actual active time (*p* = 1.06E^−13^) and number of movements (*p* = 4.42E^−2^) but not for number of movements per minute during robot-assisted therapy: the HG performed 1,023 ± 344 movements over 36 ± 3 min and the LG performed 796 ± 301 movements over 29 ± 1 min. Both groups performed 28 movements per minute. The combined program cost was €2017 and €1162 for HG and LG, respectively.

**Conclusions:**

Similar motor improvements were observed following two doses of movement-based training. The reduction in scheduled session time did not affect the intensity of the practice and met economic constraints.

## Introduction

Following stroke, around two-thirds of patients do not recover full upper limb (UL) function by the end of the subacute phase and are left with long-term disability (1). Stroke rehabilitation using robot-based upper limb therapy improves motor function and has a positive impact on activities of daily living and quality of life (2). Although is known that training intensity (i.e., rehabilitation dose) impacts neural reorganization and motor outcomes, optimal treatment doses and intensity thresholds have not been clearly established (3, 4). Repeated practice of challenging movements is key to enhance motor system connectivity and restore motor function: animal models of stroke have shown that over 400 movements per session are required for this to occur (3, 5–10). However, such a high level of repetition is not feasible within conventional rehabilitation sessions (11). Studies which attempted to achieve large numbers of movements using conventional therapy reached 289 ± 35 movements in a 1-h session; a number which is probably sub-therapeutic (12, 13). Providing additional therapy time is one solution to increase the number of movements performed; however this requires more therapists and is thus associated with higher costs. Another option is to use a robotic device. Study has shown that patients can perform 1,024 movements per 1-h session on average using a robotic device (14). The results of several controlled clinical trials have suggested positive effects of robot-assisted training programs on UL function in subacute and chronic stroke when delivered either in complete or partial substitution of conventional therapy, or in complement to conventional therapy sessions (14–19).

Our clinical practice has involved the use of a robotic device for the past 10 years. Patients perform 60 min of upper limb robot-assisted therapy (RT) per day in addition to 60 min of occupational therapy (OT). However, this mode of delivery may not be sustainable in the future: the population is aging, the number of stroke patients who require rehabilitation is increasing, health budgets are being reduced, and therapists' time is not extensible. It is therefore essential to determine the optimal practice dose which results in an increase in rehabilitation intensity and to find means to avoid increasing therapist time.

The primary aim of this retrospective study was to compare the effect of two different doses of a rehabilitation program involving combined RT and conventional OT in inpatients with subacute stroke on motor impairment. The secondary aim was to compare the costs of each dose. We hypothesized that by ensuring intensiveness within each session (using a robotic device), motor recovery would not be reduced in the lower dose group, however costs would be reduced.

## Materials and Methods

### Participants

The retrospective study was performed in accordance with current French regulation [Reference No. 004 (MR004)] and was granted approval by our internal ethics committee in line with the data protection act (20). It was registered on the Health data Hub. Two groups of patients were included: all had been hospitalized in the neurorehabilitation unit at the “Les Trois Soleils” rehabilitation Center (Boissise-Le-Roi, France) between 2009 and 2019 for stroke rehabilitation. One group followed our usual rehabilitation program (higher dose group, HG) and the second group was composed of patients who participated in a randomized controlled trial (Reference Number: ID RCB 2011-A00632-39) in our center (lower dose group, LG).

The inclusion criteria for both groups were patients: (i) with a first unilateral stroke event confirmed by computerized tomography or magnetic resonance imaging; (ii) who had undergone an UL rehabilitation program that included combined RT and OT during the subacute phase (3 weeks to 5 months post-stroke); (iii) who had completed between 20 and 35 sessions of the combined training; (iv) who had pre- and post-rehabilitation program Fugl-Meyer Assessment (FMA) scale ratings.

### Interventions

The HG underwent 60 min each of RT and OT (total 120 min per session). Within each of these sessions, 15 min were spent setting up, providing instructions, resting etc. thus practice time within the session was 45 min each for RT and for OT. Since inpatients in France usually receive 45–60 min of OT 5 days per week, we considered that RT was administered in addition to OT.

The LG underwent 35 min of OT and 35 min of RT (total 70 min per session). Within each of these sessions, 5 min were spent setting up, providing instructions, resting, etc. thus practice time within the session was 30 min each for RT and for OT. Lost time for OT and RT sessions was shorter in this group because they were performed within a clinical trial in which treatment was standardized and thus quicker to set up. We considered that in this group RT was administered in partial substitution to OT.

Robot-assisted therapy was carried out with the InMotion Arm robotic system [Interactive Motion Technologies, Inc., Watertown, MA (21)]. This two-degree-of-freedom end-effector robot trains shoulder and elbow movements in the horizontal plane using a simple visual graphical interface. Patients performed reaching movements toward visual targets in eight directions using the device. They were supervised by specifically trained therapists. The robotic system was set by the therapist to provide assistance or resistance, according to the patient's ability.

Occupational therapy sessions involved passive muscle stretching performed by the therapist, active reaching movements, grasp and release practice and functional tasks.

### Clinical Assessment

The FMA scale was used to rate motor impairment. This clinical test evaluates selective movement of the shoulder, elbow, wrist and hand (22). Ability is rated on a Likert scale, and the maximum score is 66 points. This scale is reliable and responsive for the measurement of upper-limb impairment in individuals with subacute stroke (23). In addition, the proximal (out of 42 points) and distal scores (out of 24 points) can be analyzed separately.

The mean number of RT and OT sessions completed was also recorded.

### Robot-Measured Variables

Two variables automatically recorded by the InMotion robotic arm were analyzed:

- Actual Active Time (AAT, min): average number of minutes per session when the patient actively performs movements with the robotic arm.
- Number of Movements (NM): average number of reaching movements toward visual targets in eight directions performed per session. For this variable, the training modality with the robot was not considered.

In addition, lost time was calculated by subtracting the AAT from the scheduled session time for each session, and the Number of Movements per Minute (NMM) by dividing the NPM by AAT for each session (24).

### Cost Analysis

The costs of each dose of the combined program were estimated based on the costs of RT and OT. First, the annual cost of the robotic device was calculated from the purchase value of the robot, the operating costs (maintenance, energy, consumables) and the amortization period (7 years). Calculation of the hourly cost of RT was based on use of the robot for 7 h per day, 5 days per week, for 52 weeks of the year. The hourly cost of OT was calculated in the same manner, and based on the gross annual salary (average annual gross salary of junior and senior therapists). Then, the scheduled durations of OT and RT sessions, and the level of supervision required for each were used to determine the costs of each RT and OT session. The level of supervision provided for RT was the same for both groups: one therapist for two patients (involving the cost of two robots). However, the cost differed between groups for OT. In the HG, the therapist supervised one patient for the first 30 min and two patients for the last 30 min. For the LG, the level of supervision was constant: one therapist for one patient. Finally, the average numbers of RT-sessions and OT-sessions were used to calculate the cost of the combined program for each group.

### Statistical Analysis

Baseline characteristics (age, time since stroke at the start of the program and FMA score at the start of the program) were compared between groups using a Student's *t*-test. Repeated measures analysis of variance (ANOVA) was carried out to test the effect of time (pre and post) x group (HG and LG) on FMA score. A Student's *t*-test was used to compare the number of sessions completed, AAT, NM, NMM and lost time between groups. A Student's *t*-test was also used in the cost analysis. Statistical significance was set at *p* < 0.05 and SPSS statistics 17.0 software was used for all analyses.

## Results

Thirty-six patients were included; mean (±SD) age was 62 ± 13 years, time since stroke at program initiation was 49 ± 16 days and baseline FMA score was 32 ± 12 points. Fourteen patients were included in the HG and 24 patients in the LG. Time since stroke and FMA score were similar between groups at program initiation, however, patients in the LG were older (see Table 1 for detailed characteristics).

**Table 1:** Baseline characteristics of participants.

	**HG**	**LG**
Number	14	22
Age (years)	56 ± 14	65 ± 11^*^
Female (*n*)	4	8
Side of paresis	6 R, 8 L	13 R, 9 L
Type of stroke	12 I, 2 H	20 I, 2 H
Time since stroke at program initiation (days)	49 ± 17	49 ± 17
FMA score (/66 points)	29 ± 13	34 ± 12

*Results are expressed as mean ± SD*.

*HG, higher dose group; LG, lower dose group; R, right; L, left; I, ischemia; H, hemorrhage; FMA, Fugl-Meyer Assessment*.

* *p < 0.05, T-test, HG vs. LG*.

**Figure 1:**
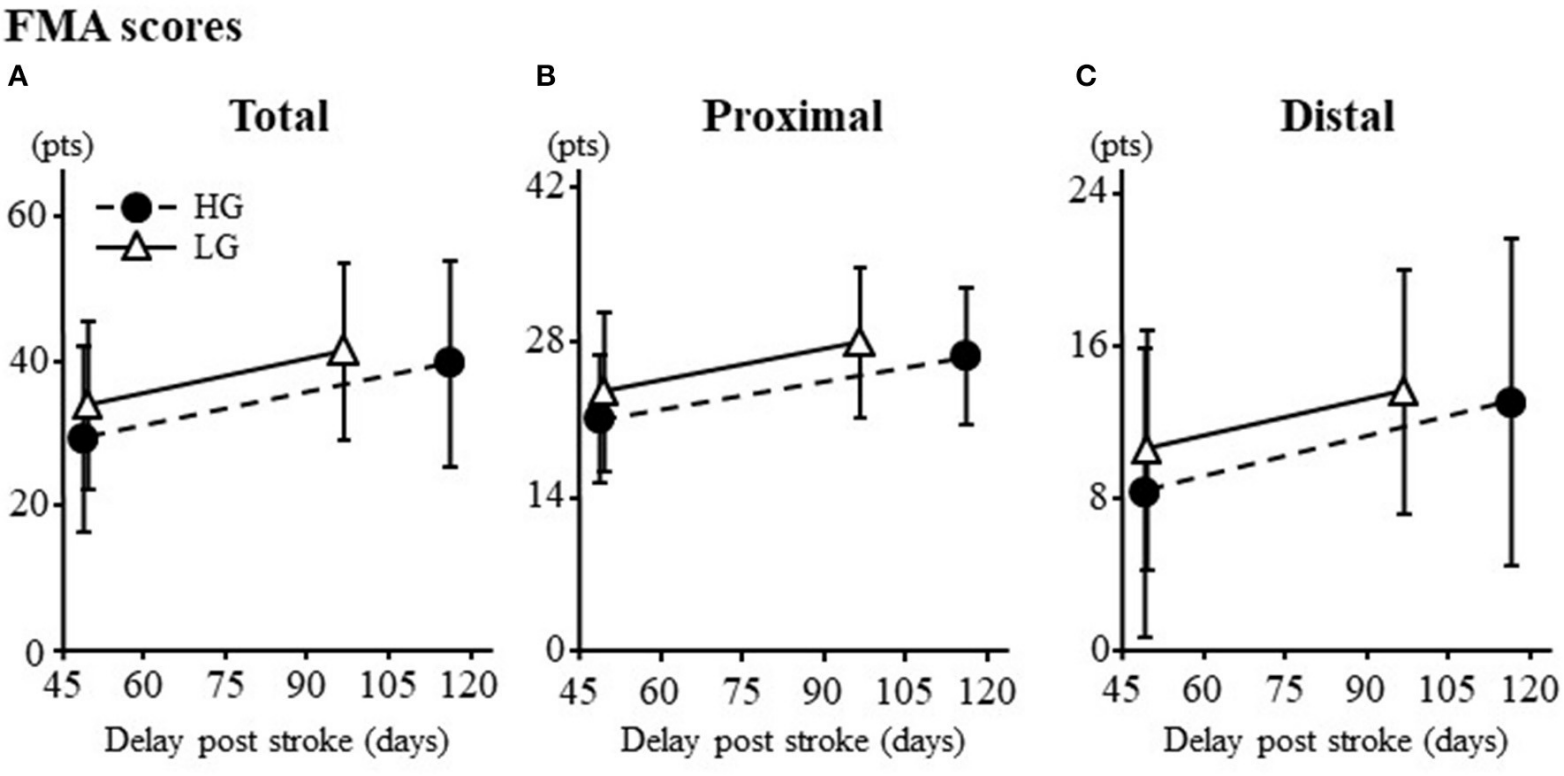
**(A)** Total score. **(B)** Proximal score. **(C)** Distal score. Results are expressed as mean ± SD. FMA, Fugl-Meyer Assessment; HG, higher dose Group; LG, lower dose Group. Maximum scores for total, proximal and distal are 66, 42, and 24 pts, respectively.

Clinical outcomes are summarized in Figure 1. Both groups improved from pre- to post-program: mean increase in FMA score was 16 ± 13% for the HG and 11 ± 8% for the LG with no between group difference in change (Figure 1A; *p* = 0.28). Furthermore, there was no difference in change between groups for proximal (Figure 1B; *p* = 0.29) and distal (Figure 1C; *p* = 0.35) FMA scores.

The results for the number of sessions completed, AAT, NM, NMM and lost time are summarized in Table 2. The mean number of sessions completed in OT (*p* = 1.37E^−3^) and RT (*p* = 1.55E^−10^), the mean NM performed in RT (*p* = 4.42E^−2^) and the mean AAT (*p* = 1.06E^−13^) were significantly higher in the HG compared to the LG. However, there was no between group difference for mean NMM (*p* = 0.88). Lost time during RT sessions was significantly higher in the HG (*p* = 5.65E^−14^).

**Table 2:** Dose parameters of the combined program.

	**HG**		**LG**	
	**OT**	**RT**	**OT**	**RT**
Scheduled session time (min)	45	45	30	30
Number of sessions completed	31 ± 2	31 ± 2	29 ± 3^*^	26 ± 2^*^
Actual active time (min)	N/A	36.0 ± 2.7	N/A	28.6 ± 0.9^*^
Mean lost time per session (min)	N/A	9.0 ± 2.7	N/A	1.4 ± 0.9^*^
Mean number of movements	N/A	1,023 ± 344	N/A	795 ± 301^*^
Mean number of movements per minute	N/A	28 ± 9	N/A	28 ± 10

*Results are expressed as mean ± SD*.

*HG, higher dose Group; LG, lower dose Group; OT, occupational therapy; RT, robot-assisted therapy*.

* *p < 0.05, T-test, HG vs. LG*.

The hourly costs of RT and OT are presented in Table 3 and the costs of the RT and OT sessions and the total cost of the combined program are presented in Table 4. The costs of both OT and RT sessions were higher for the HG (*p* = 1.49E^−15^ and 1.80E^−28^, respectively). The total cost of the combined program was higher for the HG (*p* = 6.10E^−25^).

**Table 3:** Hourly costs for a robotic device and an occupational therapist.

Robotic device	Robot purchase value	€96,394.42
	Operating costs	€24,098.61
	Amortization period (years)	7
	Annual robot cost	€17,213.29
	Effective days of use per year	235
	Daily working hours	7
	Hourly robot cost	€10.46
Occupational therapist	Gross annual salary	€59,685.42
	Effective working days per year	235
	Daily working hours	7
	Hourly cost of the occupational therapist	€36.28

**Table 4:** Session costs for robot-assisted therapy, occupational therapy and total cost for combined program.

		**HG**	**LG**
Robot-assisted therapy (RT)	Duration for RT-session (min)	60	35
	Level of supervision (therapist:patient)	1:2	1:2
	RT-session cost	€36.98	€21.57
Occupational therapy (OT)	Duration for OT-session (min)	60	35
	Level of supervision (therapist:patient)	0–30 min 1:1 30–60 min 1:2	1:1
	OT-session cost	€36.28	€21.17
Combined program	Mean number of RT-session	31	26
	Mean number of OT-session	31	29
	Mean total cost	€2,017.36	€1,162.98

*HG, higher dose Group; LG, lower dose Group*.

## Discussion

This retrospective study compared the effect of a higher and a lower dose of a combined UL rehabilitation program involving highly repetitive practice, on impairment and costs in patients with subacute stroke. In accordance with our hypothesis, there was no between group difference in change in FMA score, indicating that the lower dose of therapy induced similar changes in impairment as the higher dose. However, a control group with no RT and prospective studies on larger samples are needed to confirm this hypothesis.

The slope of the recovery recorded in the present study, in which the patients were between 1.6 and 3.4 months post-stroke, was steeper than that found for spontaneous recovery during the subacute phase of stroke (25, 26). This corroborates other findings that highly intensive therapy using a robotic device accelerates the rate of motor improvement in patients with subacute in comparison to usual care (17, 18, 27): Mean improvement in FMA score in our sample was 9 ± 7 points which are slightly higher than the typical improvement of 5 points or more with robot therapy in subacute stroke. The number of movements performed by the patients in both groups during RT in the present study (mean 1,023 ± 344 in higher dose and mean 796 ± 301 in the LG) was far higher than the number of movements usually performed in conventional OT. According to reports in the literature, a conventional rehabilitation session typically involves 32 functional UL movement attempts (11), with around 86 functional UL movements performed per day in inpatients (28). Although kinematics of movements performed are different between both therapies, the quantity completed in conventional therapy is far from being sufficient to accelerate motor recovery. Conventional therapy includes multiple therapeutic objectives, and is not only focused on practice intensity. Barriers to the provision of high-intensity arm rehabilitation related to therapists and patients need to be further examined. The results of the present study show that the number of movements performed during RT can easily exceed the thresholds required to promote of brain plasticity and motor recovery found in animal models of stroke and human motor learning studies (29, 30). Therefore, despite the shorter session time in the LG, the addition of RT to conventional OT may have helped to accelerate motor recovery during this time window by increasing treatment intensity. The results also showed that reducing the duration of the combined program did not have a negative effect on the recovery of the distal part of the UL. This could be explained by the fact that the therapists focused on the distal part of the UL during the conventional sessions since the RT involved intensive practice of shoulder and elbow movements. However, it must be noted that OT does not only involve the rehabilitation of upper limb movements and the reduction of therapy time could impact on the other aspects of this rehabilitation.

An important issue, particularly when functional scores were not evaluated, is whether the improvements were clinically important. The minimal clinically important difference (MCID) for the UL FMA is 9–10 points in subacute stroke (31). This was only achieved in the HG, probably because that group completed more sessions (i.e., their rehabilitation was evaluated over a longer time period). Since the recovery courses of both groups were perfectly parallel, it is likely that had the duration of the program been longer, the MCID for the FMA would also have been reached in the LG. An explanation that is not consistent with a previous study conducted on patient progress during upper limb robot-assisted therapy in subacute stroke subjects, using the same device. Indeed, this study had previously found that most kinematic parameters showed significant intersession differences during the first 5/10 sessions (on a total of 20 sessions) of robot-assisted therapy demonstrating that robot-assisted therapy seems to improve motor function mainly in the first sessions of treatment (32). However, we had previously shown the value on motor function of a prolonged treatment using robot-assisted therapy in an upper limb rehabilitation program (33): the short evaluation time window may have biased the interpretation of the results. Furthermore, in contrast to the mentioned study, in which the patients received only the “assisted-as-needed” robotic training modality, patients in the present study performed, according to their ability, unassisted and resistive planar reaching movements. The progression in the motor tasks' difficulty level might be a key factor in motor recovery after stroke in that it could have significantly contributed to the magnitude of motor improvement. By maintaining active patient participation, the results of this study suggest that the reduction of the duration of the combined program had a greater impact on recovery than the reduction of the session time. The rehabilitation program could be continued as long as the recovery slope does not plateau (33).

Scheduled combined session time for the LG was 33% less than the HG. Accordingly, the number of movements performed with the robotic device was significantly lower in this group; however this difference did not impact on FMA scores. Since treatment intensity (i.e., the average number of movements per minute) was the same for each group, we suggest that the intensity factor is the key influencer of patient outcomes. Another explanation for the lack of between group differences in change in FMA score is that the number of movements performed in both groups was above the threshold required to induce improvement. Indeed, the numbers performed in both groups, LG and HG, corresponded to the number of movements performed by the high intensity group in a previous study (about 750–1,000 movements per robotic session) (34). Although the threshold for the number of movements required to induce cortical changes is still unknown, several hundreds of movements of a task may be required to optimize motor recovery after a stroke. On the other hand, a ceiling effect may exist with a limited dose-response relationship once the minimum threshold has been passed (35). This hypothesis was supported by the fact all the participants in this study performed a high number of repeated movements (i.e., more than 500 movements). The results of our study therefore suggest that session time can be reduced if practice within a session is sufficiently intensive.

In addition, these results raise the issue of how to quantify intensity and dose. We named the groups in this study lower and higher dose based on the scheduled session time, however the intensity of therapy proposed was the same (i.e., number of movements/minute). The concept of treatment dose in stroke rehabilitation is complex to define, quantify and control (36). Here, we used four variables to objectively define dose: scheduled session time, number of movements performed, actual active time and number of movements performed per minute. Scheduled session time is the most common variable used to measure dose (37, 38), however, studies have shown that patients only perform physical activity for around half a typical therapy session (11, 39). In the present study, we did not attempt to quantify the time spent performing physical activity during the OT sessions, however active therapy time was measured very accurately by the robotic system during the RT sessions. In the LG, only 4 % of practice time was lost compared with 20% in the HG who received usual care. As explained in the Methods section, this is because the LG was involved in a randomized controlled trial in our center and received standardized therapy. The time lost in the HG (20%) (who received usual care) was equivalent to that reported in a previous study in which patients were found to be inactive for between 21 and 30% of a 1-h therapy session (40). Actual active time is an original variable that has rarely been used to quantify dose; according to a meta-regression analysis published in 2014, only 24% of 34 randomized controlled studies reported this variable (37). Similarly to the number of movements performed, it is difficult to measure in conventional OT but very easy in RT. Based on this parameter, patients were found to be physically active for an average of 90% (between 80 and 95%) of their RT-session duration; this is far higher than that observed in physiotherapy sessions (39). The fact that RT promotes the practice of a large number of simple movements (impairment-related therapy) in a game-like setting certainly contributed to increasing time active. Quantification of the number of movements is increasing in studies of RT and conventional therapy (11, 12, 19, 41) but if more than one type of practice is performed and/or algorithms vary, minutes of active therapy and number of movements cannot be considered as interchangeable variables. The number of movements performed per minute is a useful variable that solves this issue by controlling for differences in active therapy time across patients and type of practice. Another study of RT reported that participants performed 18 movements/minute, which is similar to the intensity in the present study (42). The choice of variable to quantify dose depends on the means and time available in the stroke rehabilitation units. In our study, the robotic device facilitated the acquisition of these variables, and in clinical practice provides therapists with a precise indication of the therapy dose received by patients.

In accordance with our hypothesis, reducing the duration of the combined program (LG) resulted in reduced costs. Importantly, this did not impact motor outcomes. Stefano et al. (43) also showed that the costs of a mixed protocol that consisted of a phase of combined robot and conventional therapy followed by a phase in which robot therapy substitutes for conventional therapy can be affordable, if the robot is easy to use and its purchase cost is reasonable. In the present study, the use of RT in partial substitution for conventional OT did not reduce the amount of motor improvement; most certainly due to the fact the robotic device provided a good tradeoff between the number of movements/session and the session duration. Providing RT in partial substitution for conventional therapy during a 1-h session therefore appears to be a useful strategy to ensure high intensity practice without increasing therapist time, and at a lower cost. In view of the many differences in the management of stroke rehabilitation units in European countries (44), this therapeutic strategy could be appropriate for health service organizations in France.

This study has several limitations. Firstly, the retrospective design means that the results may be subject to selection, implementation and evaluation bias. Secondly, in order to fully evaluate the effect of the combined program, a control group that received only conventional care would have been required. Finally, the sample size was relatively small with a heterogeneity in the number of subjects per group and in the number of treatment session. Despite the data accumulated over 10 years of routine clinical use of the robot, only a small number of patients met the inclusion criteria. However, we believe that this study provides preliminary answers to the issue of treatment dose for clinicians.

## Conclusions

This study found similar improvements in impairment following two doses of movement-based training in the subacute phase of a stroke. Robot-assisted therapy associated with conventional OT within a 1-h session is a helpful option to intensify training while meeting economic constraints.

## Data Availability Statement

The raw data supporting the conclusions of this article will be made available by the authors, without undue reservation.

## Author Contributions

CD conceived and designated the analysis. OP, TK, and A-GG collected the data. OP performed the analysis. OP and CD wrote the paper. All authors critically revised and approved the final manuscript.

## Funding

The authors would like to thank A.D.I.R.R (Association for Development and Innovation in Rehabilitation Robotics), an independent French association in providing a financial support for the overall preparation of the article (data collection, statistical analysis, interpretation of the data and writing of the report).

## Conflict of Interest

The authors declare that the research was conducted in the absence of any commercial or financial relationships that could be construed as a potential conflict of interest.

## Publisher's Note

All claims expressed in this article are solely those of the authors and do not necessarily represent those of their affiliated organizations, or those of the publisher, the editors and the reviewers. Any product that may be evaluated in this article, or claim that may be made by its manufacturer, is not guaranteed or endorsed by the publisher.
